# Low-dose valacyclovir use with preemptive monitoring in kidney transplant recipients with intermediate cytomegalovirus infection risk

**DOI:** 10.55730/1300-0144.5448

**Published:** 2022-04-19

**Authors:** Arzu VELİOĞLU, Selma ALAGÖZ, Dilek BARUTÇU ATAŞ, Hakkı ARIKAN, Ebru AŞICIOĞLU, Burak AKSU, Nurhan SEYAHİ, Serhan TUĞLULAR

**Affiliations:** 1 Division of Nephrology, Department of Internal Medicine, Faculty of Medicine, Marmara University, İstanbul, Turkey; 2 Division of Nephrology, Department of Internal Medicine, Faculty of Medicine, İstanbul University-Cerrahpaşa Faculty of Medicine, İstanbul, Turkey; 3 Department of Medical Microbiology, Faculty of Medicine, Marmara University, İstanbul, Turkey

**Letter to the Editor**,

Cytomegalovirus (CMV) infection and/or disease are among the most important infectious complications after kidney transplantation (KT). According to the recent guidelines, intravenous ganciclovir or oral valganciclovir chemoprophylaxis is recommended for KT recipients in high and intermediate risk groups [1,2]. Preemptive treatment with monitoring weekly CMV DNA without prophylaxis is an alternative approach in intermediate risk groups.

Valacyclovir has also been recommended for prophylaxis at the dose of 8 g/day for kidney transplant recipients [1,2]. Nevertheless, 3-g/day dose of valacyclovir was used in 3-kidney transplant studies that included high-risk patients and in all three studies it has been emphasized that a dose of 3 g/day provides sufficient protection for CMV [3–5]. In our institute, a modified preemptive approach, valacyclovir at the dose of 2 g/day in combination with CMV monitoring, has been used in KT recipients with intermediate risk (D+/R+) and low immunological risk. With this letter, we aimed to share our experience with a special emphasis on middle-term renal outcome, occurrence of CMV infection and/or disease and cost.

This was a two center, retrospective, observational cohort study that enrolled KT recipients from 2011 to 2017. In the study group, KT recipients have received 2-g/day valacyclovir for CMV prophylaxis with CMV PCR monitoring every 2 weeks through the first 3 months, then monthly monitoring after the third month until the sixth month. Study patients were compared with the corresponding transplant cohort from Cerrahpaşa Medical Faculty who received Valganciclovir 900 mg/day prophylaxis for 3 months during the same period. CMV PCR levels were not studied routinely in the control group, but the patients were followed up regarding CMV-specific symptoms during their regular visits. In both groups, immunosuppressive treatment protocols and follow-up protocols were similar.

A total of 62 KT recipients were included in the study (F/M: 29/33; mean age 33.4 ± 9.6; range: 18–65 years) and compared to 40 transplant recipients from Cerrahpaşa Medical Faculty. The demographic and clinical data of the study groups are summarized in Table 1. The patients’ characteristics were similar in both groups. In valacyclovir group, CMV DNAemia with a median blood level of 238 (range 141–5033) developed in 14 patients (22%) but CMV disease was not diagnosed in any of the patients. The median time post-transplant to develop CMV DNAemia was 51 days (range 11–164). In the control group, none of the patients were diagnosed with CMV disease. Late-onset CMV disease was not reported in any of the study groups. There were no reports of valacyclovir-related neurotoxicity. The incidence of leukopenia was higher in valganciclovir (Table 2). When absolute leukocyte counts at third month were compared, valganciclovir group had statistically significant lower leukocyte counts than valacyclovir group (7.1 ± 2.7 vs. 9.5 ± 3.9 10^3^/mm^3^; p = 0.001).

There was no significant difference in terms of the tacrolimus/cyclosporine use, and mean blood levels between the patients at 12th month of transplantation. Acute rejection, post-transplant diabetes mellitus, and coronary artery disease rates at the last visit were similar in both groups. There was also no significant difference with regard to glomerular filtration rate values between the groups (Table 2). Graft and patient survival rates were compared and there was no significant difference between the groups (Figures 1 and 2). The total cost of valacyclovir prophylaxis with CMV DNA PCR test was calculated as 878.52 USD whereas, in the valganciclovir group, the total drug cost was calculated as 3387.9 USD for 3 months of prophylaxis.

Although the efficacy of valacyclovir at a low dose (3 g/day) has been suggested previously, we for the first time demonstrated that the use of even a lower dose of valacyclovir (2 g/day) is also effective in KT recipients with no major adverse events and at a lower economic cost even with preemptive monitoring in every 2 weeks. Sund et al. have suggested that valacyclovir use at 3 g/day may be used for CMV prophylaxis emphasizing the need for further studies in KT recipients even though in vitro studies have demonstrated the inefficiency of low dose valacyclovir against CMV [5]. Low-dose valacyclovir prophylaxis has also been used in other immunosuppressive patient groups. An effective prophylaxis at a dose of 2 g/day has been achieved in a study on bone marrow transplantation patients [6]. In the observation of Kervan et al. on 68 patients with heart transplantation, sufficient CMV prophylaxis and a preferable cost effectiveness have been achieved with 1 g/day dose of valacyclovir [7].

In our study, CMV DNAemia frequency in the group that received low-dose valacyclovir is found to be 22%, and this rate is comparable with the previous valganciclovir studies [8,9]. In a study that investigated the efficacy of 3 g/day valacyclovir prophylaxis in KT recipients, the authors showed a 25% of CMV infection rate which was also similar to our results even we used a lower dose of valacyclovir [5]. In our study we used a modified preemptive approach, which can be defined as a combination of the two methods: using valacyclovir prophylaxis with CMV DNA monitoring. The major advantage of this combined method is that CMV monitoring would lead to early detection of breakthrough viral replication. The results of our study suggest that a modified preemptive approach may be an option in this specific group. Also, less frequent blood drawn may also result in an improvement in quality of life after transplantation.

A trend towards BK viremia was found in the study group (8% vs. 0%, p = 0.154). It can be majorly attributed to the increased rejection rate and its consequence due to increased immunosuppression in our patients. Interestingly, Reischig et al. suggested that valganciclovir prophylaxis, which generates further lymphocyte depletion, might be associated with an increased risk of BK viremia and CMV DNAemia did not appear a risk for BKV [10].

The other important issue is cost effectiveness in prophylaxis studies. The use of valganciclovir causes difficulties due to the tight monetary policies of the insurance companies in developing countries as well as in Europe and the US. It is indicated that valacyclovir even at a dose of 8 g/day has 44% lower cost than valganciclovir in the study carried out by Kacer et al. in 2015 [11]. We found that 75% lower cost was achieved in the patients who received a low dose of valacyclovir for 6 months through CMV DNA monitoring when compared to those on 3 months of valganciclovir prophylaxis.

In conclusion, our data suggest that low dose valacyclovir prophylaxis at 2 g/day with at least bi-weekly CMV monitoring for the prevention of CMV disease may have a place in patients with living kidney transplantation with intermediate CMV and low immunological risk status.

## Figures and Tables

**Figure 1:**
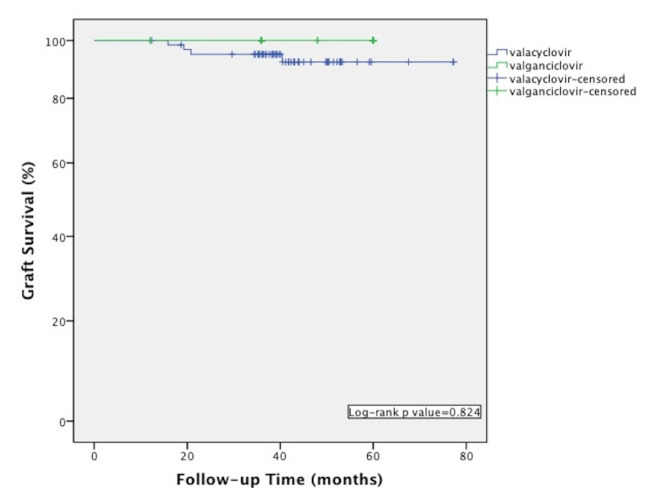
Kaplan-Meier curve showing graft survival in patients received valacyclovir and valganciclovir prophylaxis.

**Figure 2:**
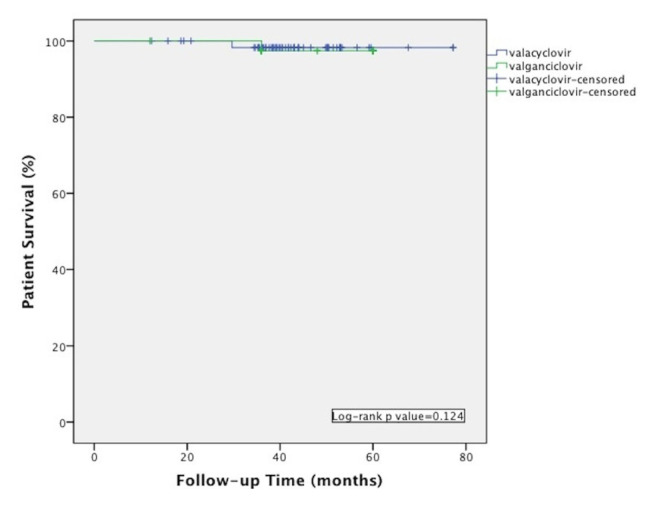
Kaplan-Meier curve showing patient survival in patients received valacyclovir and valganciclovir prophylaxis.

**Table 1:** Comparison of demographic and clinical data of study groups.

	Valacyclovir group (n = 62)	Valganciclovir group (n = 40)	p

Gender (Female/male)	29/33	14/26	0.227

Age (years)	33.4±9.7	36.6 ±13.5	0.163

Primary kidney disease n (%)			0.063
Glomerulonephritis	20 (30.6%)	8 (20%)	
Diabetic nephropathy	7 (11.3%)	5 (12.5%)	
Hypertension	3 (4.8%)	1 (2.5%)	
Reflux nephropathy	4 (6.5%)	11 (27.5%)	
Polycystic kidney disease	4 (6.5%)	0 (0%)	
Unknown	24 (38.7%)	15 (37.5%)	

Hepatitis serology n (%)			0.472
Hepatitis B	4 (6.5%)	1 (2.5%)	
Hepatitis C	1 (1.6%)	0 (0%)	

Immunosuppressive drugs n (%)			0.390
Tacrolimus	57 (91.9%)	38 (95%)	
Cyclosporine	5 (8.1%)	2 (5%)	

HLA mismatch number (MM)			0.512
0–1 MM (n)	15	7	
2–4 MM (n)	34	21	
5–6 MM (n)	13	12	
Donor sex (F/M)	39/23	29/11	0.391

Donor age (years)	48.5 ± 9.6	45.9 ± 10.4	0.204

**Table 2:** Clinical outcomes of study groups.

	Valacyclovir group (n = 62)	Valganciclovir group (n = 40)	p
GFR (mL/min/1.73 m^2^)			
GFR at 1 month	71.4 ± 19.2	75.2 ± 21	0.349
GFR at month 3	69.1 ± 21.5	74.3 ± 16.9	0.195
GFR at month 6	73.8 ± 22.3	71.3 ± 16.2	0.557
GFR at month 12	69.1 ± 22.8	74.6 ± 19	0.202
GFR at last visit	63.9 ± 25.2	71.4 ± 20.4	0.119
Hgb (g/dL)			
Hgb at 3 months	12.9 ± 1.6	12.7 ± 1.3	0.516
Hgb at 6 months	13.5 ± 1.8	13.2 ± 1.5	0.350
Wbc (10^3^/mm^3^)			
Wbc at 3 months	9.5 ± 3.9	7.1 ± 2.7	0.001*
Wbc at 6 months	8.5 ± 3	7.6 ± 2.1	0.095
Leukopenia at 3 months (n, %)	6 (9.7%)	8 (20%)	0.153
Mean tacrolimus levels (μg/L)	8.8 ± 1.1	9.4 ± 1.4	0.095
Mean cyclosporine levels (μg/L)	132.8 ± 17	136 ± 34.3	0.862
Acute rejection**	10 (16.1%)	4 (10%)	0.557
Follow up time (months)	42.7 ± 12.1	43.2 ± 12.1	0.861
Posttransplant DM**	4 (6.4%)	8 (20%)	0.057
BK viremia**	5 (8%)	0 (0%)	0.154
Cardiovascular event**	0 (0%)	3 (7.5%)	0.058
Graft loss**	4 (6.4%)	0 (0%)	0.153
Death**	1 (1.6%)	1 (2.5%)	1

* Statistically significant, p < 0.05;

** At the end of follow-up.

GFR: Glomerular Filtration Rate; Hgb: Hemoglobin; Wbc: White blood cell; DM: Diabetes Mellitus.
